# An exploratory non-randomized study of a 3-month electronic nicotine delivery system (ENDS) intervention with people accessing a homeless supported temporary accommodation service (STA) in Ireland

**DOI:** 10.1186/s12954-020-00406-y

**Published:** 2020-10-12

**Authors:** Florian Scheibein, Kevin McGirr, Andy Morrison, Warren Roche, John Stephen Gary Wells

**Affiliations:** 1 School of Health Science, Waterford Institute of Technology, Waterford, Ireland; 2 University of California San Francisco, San Francisco, USA; 3 New Nicotine Alliance, London, UK; 4 Nutritional Research Centre Ireland, Waterford Institute of Technology, Waterford, Ireland

## Abstract

**Background:**

Smoking is endemic amongst people accessing homeless services, and they are disproportionately affected by smoking-related diseases. This paper reports on the results of a 3-month small scale intervention which explored the efficacy, challenges and opportunities of using electronic nicotine delivery systems (ENDS) to support cessation of tobacco smoking with people accessing an Irish supported temporary accommodation (STA) homeless service. It considers the results of this intervention with reference to the balance of harms between the use of vaping to support smoking cessation and continued smoking.

**Methods:**

Twenty-three participants were recruited. Demographic data, carbon monoxide (CO) measurements, homelessness status and smoking history were recorded. Participants were given an ENDS device and two 10-ml bottles containing e-liquid available in several flavours and at several strengths. Participants could pick up new bottles on a weekly basis. At weeks 1, 4, 8 and 12, the Fagerström Test and Mood and Physical Symptoms Scale (MPSS) were administered.

**Results:**

Over 75% of the residents in the participating hostel were recruited (23/30). However, there was a substantial loss to follow-up (*n* = 14) as a result of data protection issues, the transient nature of the population of interest and non-compliance with the intervention.

Self-reported reductions in cigarette consumption were found to be statistically significant (*p* < 0.001). However, reductions in carbon monoxide measurements were not statistically significant. Decreases in Fagerström Nicotine Dependence Test were statistically significant (*p* = 0.001), but decreases in MPSS “urge to smoke” and “strength of urges” composite scores were not.

Reported side effects included coughing, runny nose, bleeding nose, slight sweating, dizziness, increased phlegm and a burning sensation at the back of the throat. Barriers to engagement were peer norms, vaping restrictions in accommodation and adverse life events.

Positive effects reported included increased energy, less coughing, better breathing and financial benefits. An improvement in the domain “poor concentration” was also found to be statistically significant (*p* = 0.040).

**Conclusion:**

ENDS-based interventions may be effective with this population. Future research should aim to improve follow-up, consider including behavioural components and monitor health effects in relation to ongoing concerns around risks and the balance of harms.

**Trial registration:**

Registered retrospectively ISRCTN14767579

## Background

A stressful social environment where tobacco smoking is often normalised can make it difficult for people using homeless services to quit tobacco smoking or to remain abstinent [1]. Tobacco smoking is widespread amongst homeless populations [2, 3], who are disproportionately affected by tobacco-related diseases such as chronic obstructive pulmonary disease—COPD [4]. Recent studies indicate that people accessing homeless services can be receptive to approaches involving electronic nicotine delivery systems (ENDS) [4, 5]. A pilot study by Collins et al. [5] used an approach that combined education, counselling and the provision of an ENDS device to a population accessing homeless services and found this combined approach to be effective [6].

Preliminary evidence suggests that ENDS-based interventions may be more effective than nicotine replacement therapy (NRT) and other common medications such as varenicline and bupropion for smoking cessation in the general population [7, 8]. When reported side-effects were compared between people using NRT or e-cigarettes, throat or mouth irritation was reported more frequently in the e-cigarette group (63.5% vs 51.2%) [7]. However, the e-cigarette group reported greater declines in the incidence of cough and phlegm production from baseline (relative risk for cough, 0.8; 95% CI 0.6 to 0.9; relative risk for phlegm, 0.7; 95% CI 0.6 to 0.9), and there was no statistically significant differences between incidence of wheezing or shortness of breath (ibid.).

A number of concerns have been raised around using ENDS products long term including the potential development of e-cigarette or vaping product-associated lung injury (EVALI) [9–12] and potentially harmful effects on the cardiovascular system [9]. However, EVALI appears to be linked to the additive vitamin E acetate [10, 11]—an adulterant associated with black market-sourced cannabinoid oil samples [12] and Public Health England considers ENDS-devices 95% safer than tobacco smoking [13].

ENDS-based interventions appear to have a relatively benign side-effect profile when compared to some of the recommended treatments for tobacco dependence such as varenicline, bupropion and nicotine replacement therapies [7, 8]. Concurrently, whilst ENDS devices are associated with risks [8–10, 14], switching from tobacco smoking to ENDS devices "could help reduce smoking related disease, death and health inequalities" [13]. Evidence suggests that ENDS-based interventions may be helpful in helping people accessing homeless services to give up smoking tobacco [4, 5]. However, the efficacy, challenges and opportunities of such approaches have not been studied in relation to homeless supported temporary accommodation (STA) services specifically. Furthermore, there has been little discussion of the balance of harms between using ENDS interventions and smoking in light of the recent controversies and concerns around “vaping” products. When this study was initiated, this controversy did not exist. However, we consider these issues in relation to the results we report here. Thus, this study sought to explore these domains with reference to a 3-month ENDS-based intervention (March to June 2019).

## Methodology

Participants were recruited in a STA service in Ireland (*n* = 23). STAs are services which generally provide a room for a period of 6 months for people registered as homeless. This STA is a 30-bed service with a mix of private and shared rooms with a number of private rooms for couples. Smoking is prohibited indoors according to national legislation. No such ban applies to vaping. All meetings with study participants in the STA occurred in the on-site nurse station and more rarely in an on-site meeting room.

STA project workers and support staff identified potential study participants who smoked and wished to quit. They provided them with an information leaflet (see https://osf.io/rq82w/) and informed them of the date and time when the lead researcher would visit the service. The lead researcher also discussed the study with potential study participants when asked to do so by attendees of the service. Meetings were arranged for each of the potential study participants where the details of the study were explained to them. Signed informed consent forms (see https://osf.io/92wz6/) were obtained from those willing to participate in the study. One-to-one in-person meetings took place in most cases. However, meetings with couples were also conducted as this was preferable to some study participants. Enrolled study participants were encouraged to let their peers know about the study and how to participate if they wanted to do so.

Participants were asked to indicate their active smoking status and whether they wished to give up smoking. Individual participants needed to score > 5 ppm (parts per million) CO (carbon monoxide) as measured using the Smokerlyzer® device to meet the inclusion criteria for this study. Previous research suggests that those measuring < 5 ppm CO are unlikely to be smokers [15]. Participant exclusion criteria included self-reported pregnancy and the exhibition of florid psychotic or substance use-related symptoms which could have affected ability to consent.

At week one, demographic data, homelessness history and smoking history were recorded. In addition, participants completed the Fagerström Test for Nicotine Dependence and the Mood and Physical Symptom Scale. The Fagerström Test is a well-established 6-item measure for nicotine dependence. The Mood and Physical Symptom Scale is a widely used 12-item measure often used to check for nicotine withdrawal symptoms. It is divided into three parts: mood symptoms, urge to smoke/intensity of urge and physical symptoms. Both tests were adjusted for smoking only.

Participants were given an electronic nicotine delivery system device (Endura T22e™) and 2 × 10-ml nicotine-containing fluid vials available in a variety of strengths (0, 6, 11, 18 and 20 mg/ml) and flavours (berry, menthol, regular tobacco, American tobacco). The Endura T22e™ starter kit was selected for its durability, ease of use, long battery life and because it is a "mouth to lung" device which better emulates the senation  of smoking compared to "direct to lung" devices.  The Endura T22e™ conforms to European Tobacco Productive Directive requirements and is available commercially in Ireland. The model is also used in an ongoing Australian clinical trial with people living with HIV (ACTRN12616001641482) and another with people engaging with substance use treatment [14]. The model consists of a tank which screws into the battery. This holds the fluid, which is then vaporized. It is a low maintenance midrange device, and the ongoing costs (coils, liquid, etc.) are much lower than pod-based or single use systems. Despite its relative low cost, affordability for people accessing homeless services may be a factor for its use, and indeed in this study, the device was fully subsidized. Where possible, broken or stolen devices were repaired or replaced.

Participants were compensated for their time with a 15-euro “One4All®” voucher at weeks 1, 4, 8 and 12. Financial incentives were included as previous research found that “...financial incentives added to free cessation aids resulted in a higher rate of sustained smoking abstinence than free cessation aids alone” [16].

Number of cigarettes smoked, CO measurements, Fagerström Test scores and MPSS scores were recorded at weeks 1, 4, 8 and 12. These meetings were considered mandatory for the successful completion of the study. Participants had the option to meet the primary researcher weekly at the service for further allotments (2 × 10 ml) of vaping fluid and support. During all sessions CO measurements were obtained, and participants discussed any problems with the device and any benefits or side-effects experienced. All reported side-effects and positive effects were recorded.

In response to a dropout rate of over 20%, we adopted a per protocol approach to prevent false positive results [17]. All analysis was carried out through Minitab® 17.2.1, and a 5% level of significance was applied. No adjustments were made for multiple comparisons as this would reduce the power of the study further. In order to assess statistical significance, we ensured that the assumptions of the 2-sample *t* tests were met. For data which was not normally distributed at both baseline and end-of-study time points, statistical significance was not reported.

A Pearson correlation test was used to assess the relationship between continuous variables (such as age and number of cigarettes smoked), and Spearman correlation was used to assess the relationship between interval data (for example, to assess the relationship between difference in number of cigarettes smoked and years smoking). Boxplots, interval plots and scatter plots were used as necessary for visual representation of data. For each interval plot discussed, a 95% Confidence Interval is constructed around each mean value.

## Results

Of 30 STA residents, 23 enrolled. In total, 14 recruited participants out of the 23 did not complete the intervention, leaving 9 participants who did. Two identified as women and seven identified as men. Study participants were aged between 32 and 54 years old (mean 43.89 years; SD 7.36 years), first became homeless between 15 and 50 years old (mean 35.22 years; SD 13.07 years) and had been homeless between 1 and 22 years (mean 7.33 years; SD 5.87 years). Study participants reported starting smoking between 7.5 and 18 years (mean 13.17 years; SD 2.98), having smoking histories between 14 and 36 years (mean 30.44; SD 9.37) and smoking between 12 and 30 cigarettes per day (mean 25.22; SD7.77). At baseline, study participants measured between 7 and 53 ppm CO (mean 21.89; SD 13.59), between 7 and 9 in the *Fagerström* (mean 7.8; SD 1.2), between 7 and 26 in MPSS mood symptom composite score (Q1–7) (mean 18.11; SD 8.13), between 3 and 10 in MPSS “Urge to Smoke” and “Strength of Urges” composite score (Q8 and 9) (mean 6.89; SD 2.69) and between 3.55 and 4.55 in the MPSS physical symptom composite score (mean 4.56; SD 1.5) (see Table 1).

**Table 1:** Demographic and baseline data (*n* = 9)

Variable	Mean (SD)	Range
Age	43.89 (7.36)	32–54
Age first homeless	35.22 (13.07)	15–50
Years homeless	7.33 (5.87)	1–22
Age first smoked	13.17 (2.98)	7.5–18
Years smoked	30.44 (9.37)	14–36
Number of cigarettes smoked	25.22 (7.77)	12–30
Carbon monoxide (ppm)	21.89 (13.59)	7–53
Fagerström	7.89 (1.2)	7–9
MPSS (Q1-7)*	18.11 (8.13)	7–26
MPSS (Q 8 and Q9)**	6.89 (2.69)	3–10
MPSS (Q10-12)***	4.56 (1.5)	3.55–4.55

*Composite score of mood symptoms

**Composite score of “Urge to Smoke” and “Strength of Urges”

***Composite of physical symptoms

One participant only used the device “when there is no tobacco”, and eventually stopped using the device entirely because it could not mimic the effect of smoking tobacco efficiently. When asked to describe this, he said:

“No tobacco taste. It doesn’t hit the ‘gspot’ at the back of the throat. If it would taste like a cigarette it would be great”. He was not included in the statistical analysis.

Of the nine participants who completed the intervention, the following flavours were indicated as the most popular: “Purple Berry” with either “Regular Blend” (British tobacco flavour) or “American Tobacco” (*n* = 4), “Purple Berry” only (*n* = 2), “Regular Blend” (*n* = 2) and “American Tobacco” (*n* = 1). Although some participants indicated a preference for menthol, none of these completed the study.

### Efficacy

**Fig. 1:**
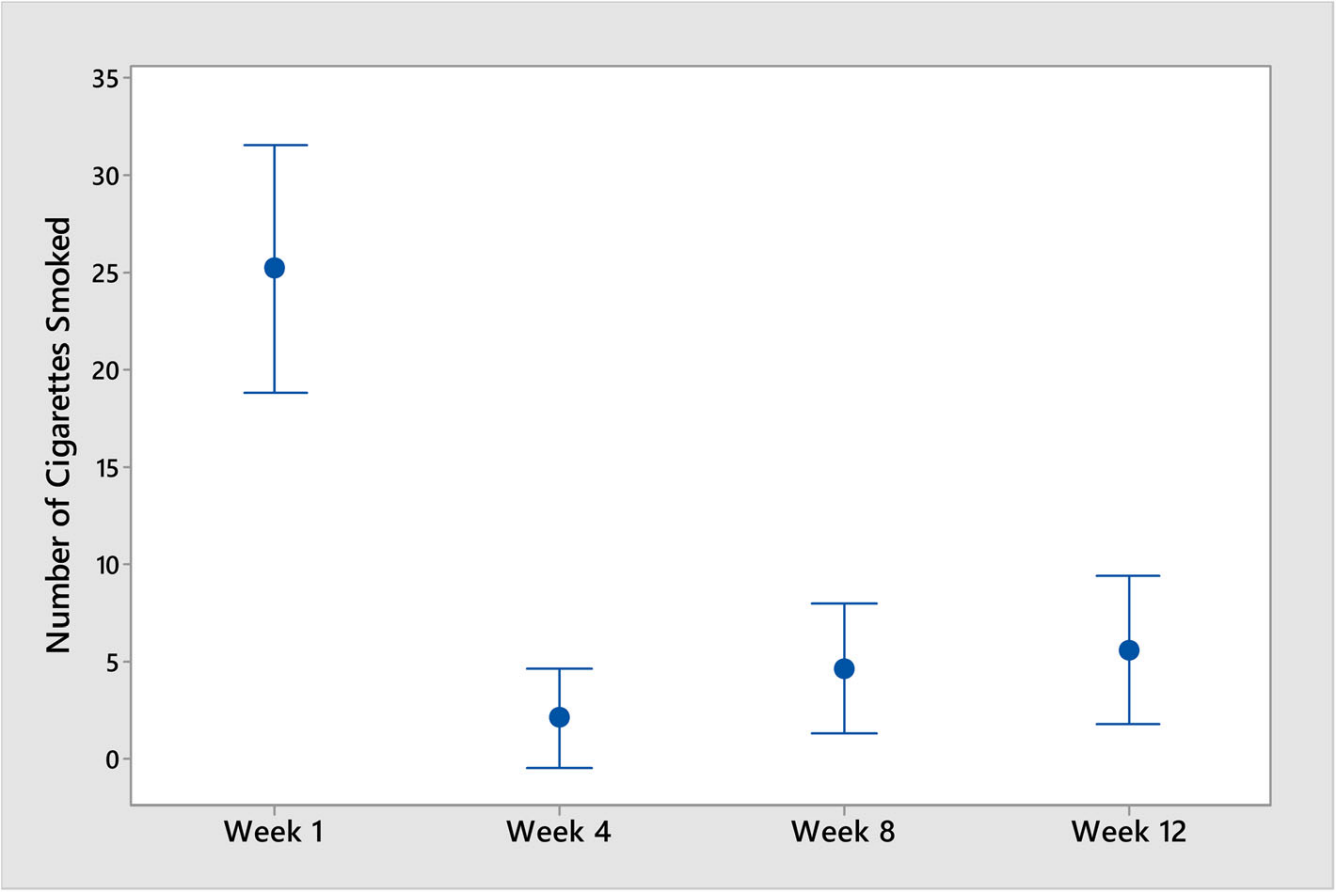
Interval plots of cigarettes smoked

**Fig. 2:**
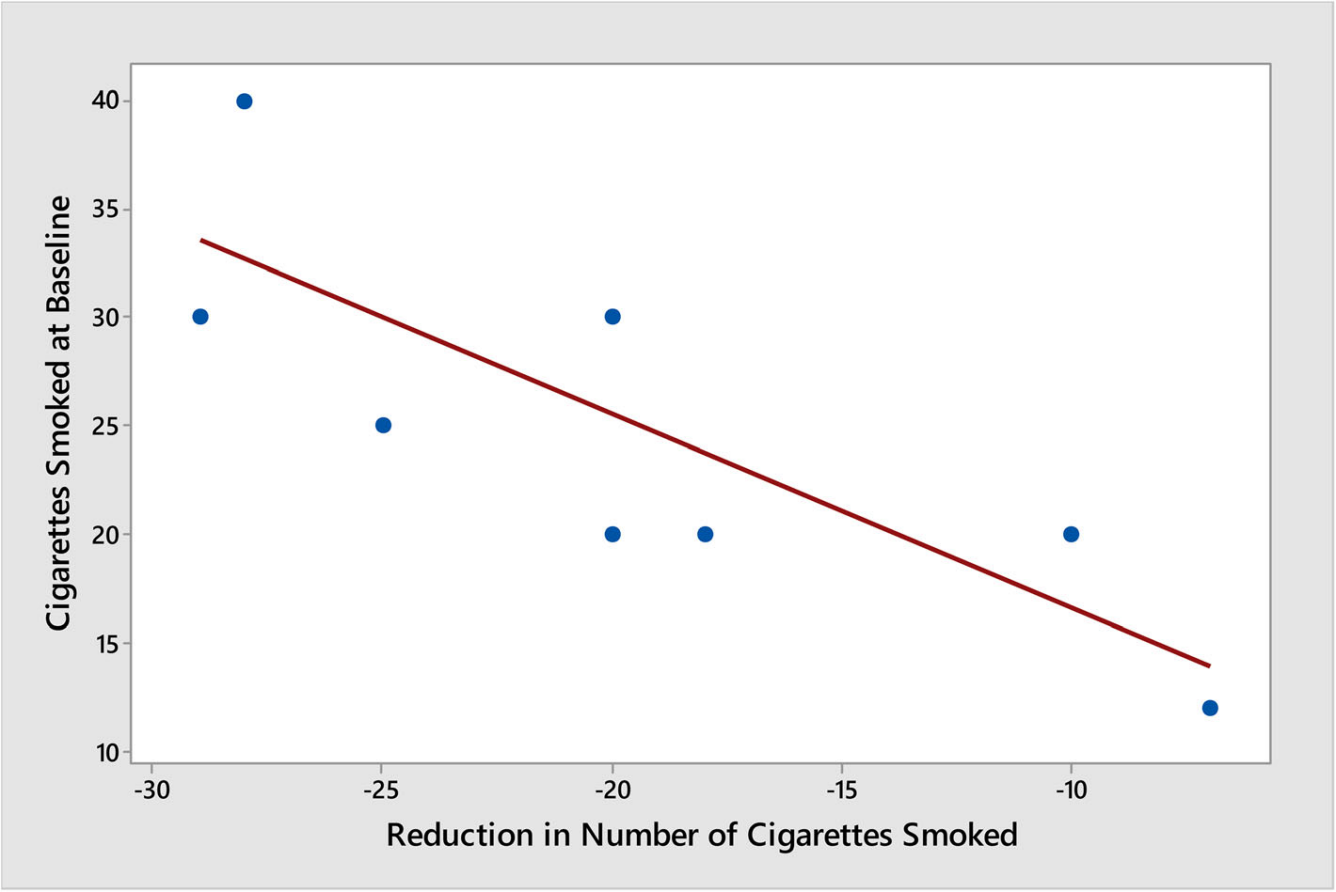
Cigarettes smoked at baseline vs reduction of cigarettes smoked

Cigarette consumption was reported to decrease from a mean of 26.7 to 9 cigarettes (75% reduction). This self-reported decrease of cigarettes smoked was statistically significant (*p* < 0.001) (see Fig. 1). Mean carbon monoxide measurements decreased from 21.3 to 16.1 ppm CO (35% reduction) with one participant measuring below the 5 ppm CO requirement to be considered a smoker. However, this decrease was not statistically significant (*p* = 0.472). The higher the number of cigarettes reported to be smoked at baseline, the lower the number of cigarettes decreased (*p* = 0.009; *r* = − 0.807) (see Fig. 2). However, there was no statistically significant relationship between the number of cigarettes smoked at baseline and reductions in carbon monoxide (*p* = 0.472). Furthermore, there were no statistically significant relationships between years homeless, years smoked or “Quit” attempts and reductions in cigarette smoking.

**Fig. 3:**
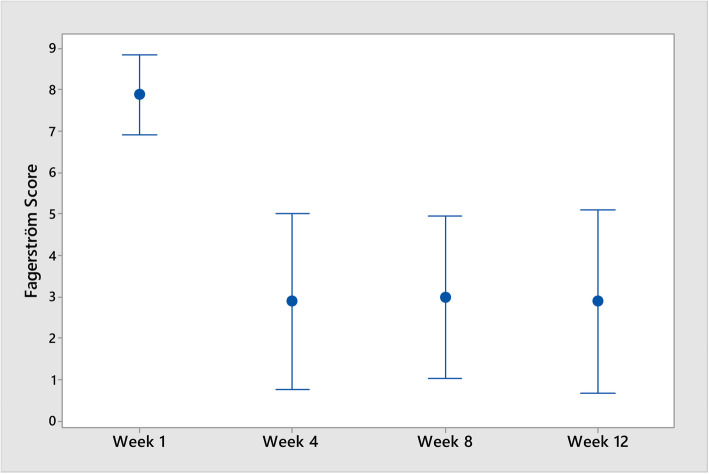
Interval plot of Fagerström scores

**Fig. 4:**
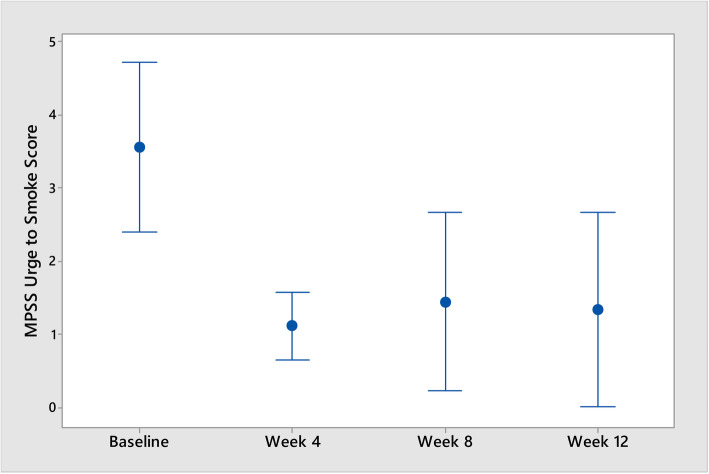
Interval plot of urge to smoke

**Fig. 5:**
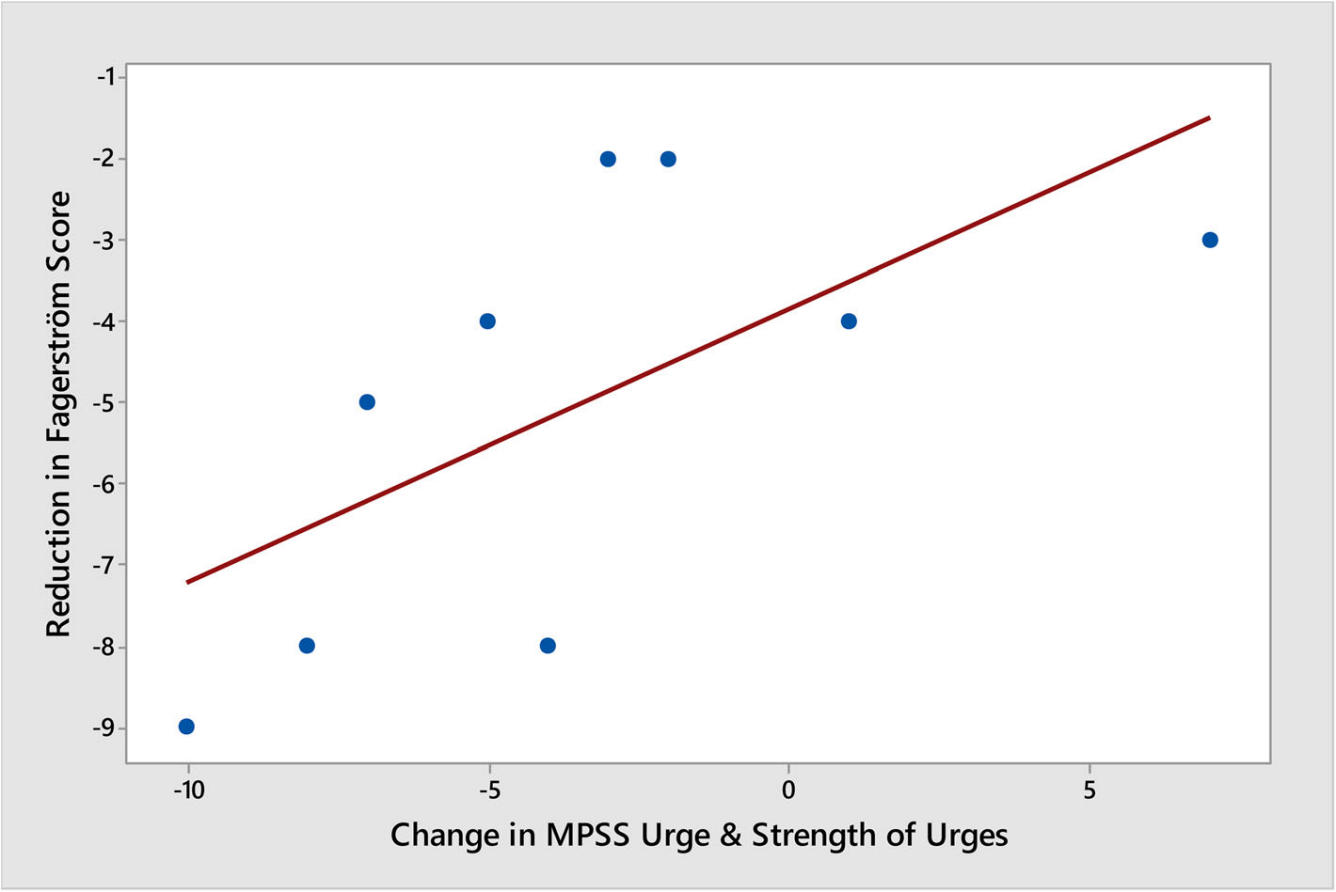
Reduction in Fagerström vs change in MPSS urge and strength

The decrease in Fagerström Test Scores between baseline and week 12 was statistically significant (*p* = 0.001) as was a decrease in “urge to smoke” (*p* = 0.030). Neither decreases in the MPSS “strength of urges” domain or the composite score over the trial period were statistically significant at *p* = 0.216 and *p* = 0.079, respectively. However, the average decreases in the Fagerström Test Scores against the averages decreases in the MPSS composite score, over the trial period, were statistically significant (*p* = 0.018; *r* = 0.760) (see Figs. 3, 4, and 5).

### Life stresses and device problems

Three participants reported using cigarettes as a coping mechanism for stressful life events (for example, deaths, hospitalizations of partners and family members) whilst one participant reported using smoking to cope with life stresses, for example struggling to pay rent. Smoking levels were reported to increase during these times and normalise again over time.

Smoking peer norms and life pressures (such as stresses related to homelessness) were identified as barrier to stopping smoking. One participant characterised smoking as being “part of the lifestyle of drugs” associated with homelessness, whilst another participant found it difficult to stop smoking “at night-time, when you drink”.

In one case, a participant, who described the device as “a fantastic aid”, moved to a new service where there was a “vape ban”. This vape ban led to the exposure of the participant to peer smoking norms in an outside smoking area. This may have contributed to his relapse to cigarette smoking. As he said:

When you have to get up to get out to vape you usually end up having to smoke. People end up getting you to smoke. I don’t know if it’s their guilty conscience or I look sad. It’s like being forced to go to the pub. I could drink 2-3 orange juices but I’m not likely to.

Participants reported several losses, breakages and thefts of the vape device. For example, one device was accidentally dropped into water, and others broke because the tanks were not correctly attached. These breakages were easily resolved, in most cases, by drying the battery and/or unscrewing and replacing the tanks.

### Physical and psychological impacts of the intervention

Several participants (6/9; 67%) reported negative physical side effects including runny nose (*n* = 1), bleeding nose (*n* = 1), slight sweating and dizziness (*n* = 1), increased phlegm (*n* = 1), burning sensation (*n* = 1) and coughing (*n* = 3). In one case, a person living with COPD had an acute coughing fit as a result of using the device for the first-time. However, two people volunteered information that they had COPD, and both reported that their health improved during the study period.

Several participants (4/9; 44%) also reported positive physical improvements. These included breathing better (*n* = 3), less coughing (*n* = 1), being more “active” (*n* = 1) and having more “energy” (*n* = 1).

**Fig. 6:**
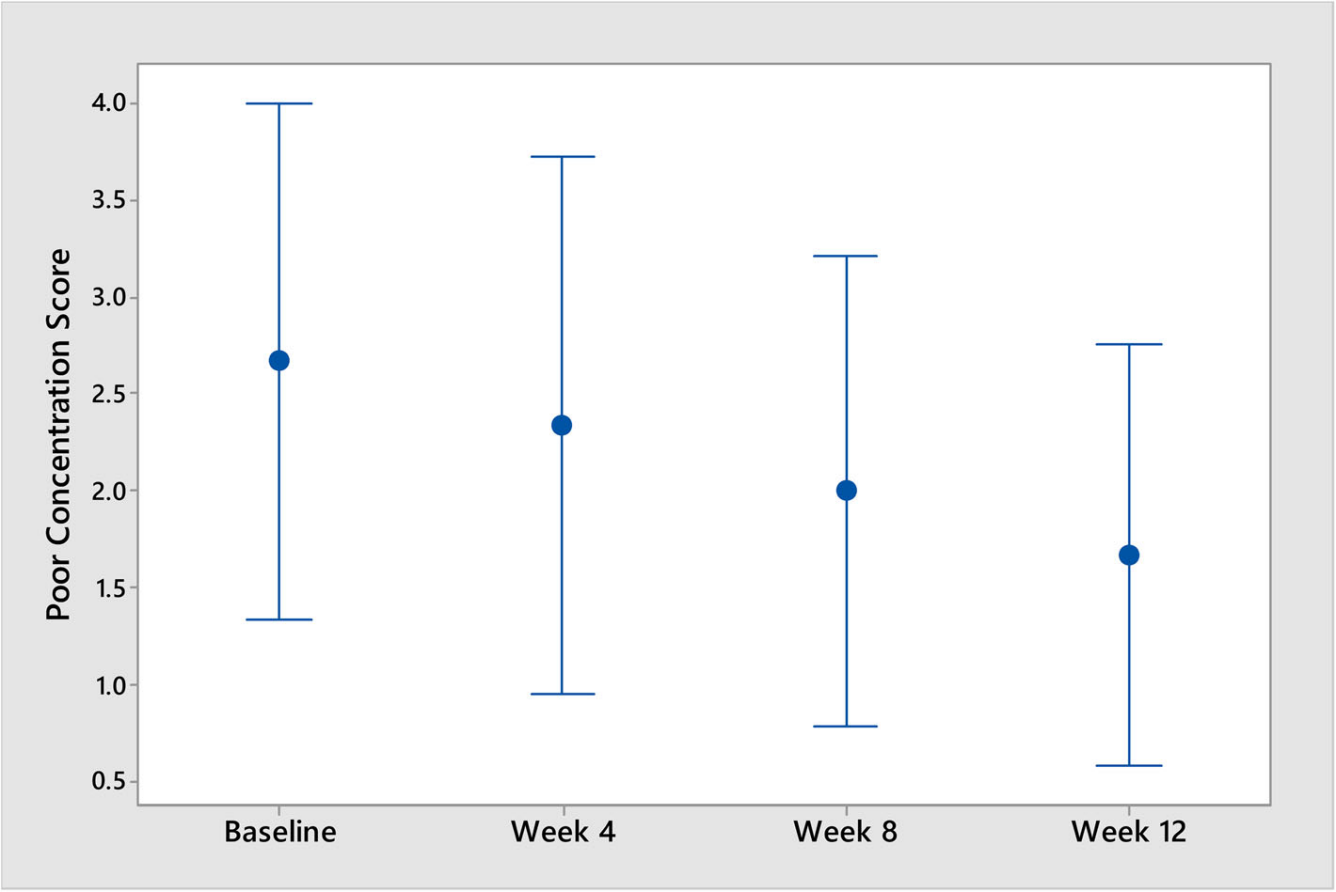
Interval plot of poor concentration

The MPSS is used to monitor adverse effects of nicotine withdrawal. However, since this intervention used a nicotine-based intervention, it was unclear what if any effect the intervention would have on this domain. Overall mood symptoms (depression, anxiety, irritability, restlessness, hunger, poor concentration and poor sleep) decreased during the trial period but this reduction was not statistically significant (*p* = 0.193). Reductions in the parameter “restlessness” from baseline to week 12 were also not statistically significant (*p* = 0.094), but improvements in the parameter “poor concentration” were statistically significant (*p* = 0.040) (see Fig. 6).Within this context of psychological improvements, one participant also reported an improvement in her relationship with her son as a result of taking part in the intervention.

### Motivations for engaging with the intervention

Two participants reported financial benefits as a motivator for engagement with the study. The participant said:

I must have spent a fortune on cigarettes. When I got the chance of getting a vape I jumped at it.

Another participant, who had both COPD and lung cancer, also suggested that moving out of homelessness might be an opportune moment to engage in such interventions as it removed the pressure of peers to smoke in his environment:

Deadly. Very good. If I was in a house on my own I would be off the cigarettes altogether.

## Discussion

The initial high uptake within the study suggests that people accessing STAs are keen to engage with tobacco harm reduction interventions [5]. The intervention led to reductions in self-reported tobacco smoking amongst the participants who completed it (*n* = 9). This was biochemically validated (with reductions in CO ppm), but these reductions did not reach statistical significance due to the low sample size and potentially due to secondhand exposure to tobacco smoke in shared spaces (many participants shared a room with one or more smokers). Two participants reported complete abstinence at the end of the intervention period. Notably, they had increased their socioeconomic status (SES) through part-time employment and moving out of homelessness respectively.

Our findings add to evidence that housing, employment and other forms of SES may be predictive of the success of smoking cessation attempts [18]. These findings also highlight the potential role of homelessness, unemployment and other forms of socioeconomic disadvantage as key drivers for smoking-related behaviours. Future research should consider ENDS-based interventions in the context of other interventions which aim to increase SES such as housing or employment.

It is likely that picking a “mouth to lung” device, which more closely resembles the act of smoking than “direct to lung” models, and including both fruit and tobacco flavours were instrumental in the initial high acceptance of the intervention. Making the right choice of product in relation to the target population illustrates the importance of consumer involvement in tobacco harm reduction research [19]. It should also be noted that the flavours of vaping products may affect participant engagement with the intervention with one participant dropping out of the study because of the taste.

This intervention was a pharmacological one. Participants could drop in for more nicotine-fluid as needed. It did not include any behavioural component such as motivational interviewing, cognitive behavioural therapy, counselling or other evidence-based psychological intervention. This was regrettable as evidence suggests that e-cigarettes are more effective than nicotine replacement therapy when both are combined with behavioural support [7]. Future studies should include such supports as part of the intervention design; though this is likely to increase the cost of the intervention.

Several study participants reported that stressful life events (bereavements, hospitalisations) and life stressors (for example, the inability to pay rent) were a barrier to stopping smoking. This is consistent with the literature that life events and stress drive smoking [20–22].

Future research should be cognizant of the inherently stressful nature of the experience of homelessness [1] and aim to provide a more holistic range of supports, such as stress management and coping skills if desired.

Limited negative side effects possibly related to vaping (for example, a runny nose, a nosebleed, slight sweating, coughing and increased phlegm) were reported. This confirms literature that suggests that ENDS devices are well tolerated when compared to NRT [6]. However, there was an acute coughing episode in a participant who lived with COPD suggesting that extra precaution is required when introducing people with COPD to vaping.

The limitations to this study include a small sample size (*n* = 9), a high loss to follow-up, a lack of behavioural support and a lack of health information. One reason for the high loss of follow-up was that many participants transitioned from the service during the intervention period. The primary researcher obtained written consent from participants to obtain information on transfers to other services. However, service providers did not have written consent from participants to supply this information to the primary researcher. This led to a situation whereby service providers could not supply information concerning the residency status of participants as it would breach newly enacted European General Data Protection Regulations (GDPR). Researcher follow-up, therefore, was only possible with participants who gave advance notice of transitioning to other services. Ability to follow-up was further complicated by the fact that weekly meetings were voluntary, so sometimes long periods elapsed between contact times. In total, the primary researcher met three participants at three different services outside the study site, but most participants who moved to other services were lost to follow-up.

Follow-up may have been improved with weekly drop-in groups focused on vaping and trouble shooting in relation to problems. The low overall sample size that statistically significant inferences, viz. generalisable suggests recommendations to this population need to be treated with caution and may not be appropriate. We also chose to use a “Per Protocol” approach as the large dropout rates would have likely contaminated the data with false positives in an intention to treat analysis [17]. We could not obtain baseline health data due to data protection restrictions set by the Ethics Committee. This data would have provided additional information that might have provided greater insight in relation to the findings. Our study design also did not include a behavioural component. Such a component could have increased the efficacy of the intervention.

The World Health Organization advises caution with regards the potential harms of ENDS devices [23]. However, the risk of ENDS can be set against the relative risk of such products as combustible and heated tobacco [13]. When such harms are ranked, a group of experts determined that the harms associated with combustible tobacco were the largest, the harms associated with NRT the lowest and the harms of ENDS devices above NRT but lower than heated or combustible tobacco [24].

Whilst the long-term harms of ENDS are unknown, they are likely to be lower than those associated with combustible tobacco [13]. However, there may be significant harms associated with ENDS devices in particular cases [12] for example people who are allergic [15] or those with pre-existing lung conditions as found in this study.

Evidence suggests that ENDS devices can be used effectively in short-term interventions to reduce tobacco smoking with limited side effects [6, 7]. This includes people accessing homeless services [4, 5] which are highly likely to smoke and disproportionately experience smoking-related harms [2]. This study adds to the evidence that ENDS devices may be useful in helping such individuals to reduce or stop smoking tobacco as part of structured interventions.

## Conclusions

This intervention was found to reduce self-reported tobacco smoking in a population accessing homeless services. Both study participants who reported complete tobacco smoking abstinence had recently increased SES in the form of transitioning out of homelessness and gaining employment, respectively. This warrants future research into the potential benefit of recently accumulated SES in the success of similar interventions. Improvements in mood (particularly in terms of concentration) should be considered in future research as the mechanism of this statistically significant finding is unclear.

## Abbreviations

ANDS: Alternative nicotine delivery systems
ENDS: Electronic nicotine delivery system
STA: Supported temporary accommodation
