# Correction to: An exploratory non-randomized study of a 3-month electronic nicotine delivery system (ENDS) intervention with people accessing a homeless supported temporary accommodation service (STA) in Ireland

**DOI:** 10.1186/s12954-021-00549-6

**Published:** 2021-11-09

**Authors:** Florian Scheibein, Kevin McGirr, Andy Morrison, Warren Roche, John Stephen Gary Wells

**Affiliations:** 1 School of Health Science, Waterford Institute of Technology, Waterford, Ireland; 2 University of California San Francisco, San Francisco, USA; 3 New Nicotine Alliance, London, UK; 4 Nutritional Research Centre Ireland, Waterford Institute of Technology, Waterford, Ireland

## Correction to: Harm Reduction Journal (2020) 17:73 10.1186/s12954-020-00406-y

Following publication of the original article [1], an error was identified in the Table 1 and in the first paragraphs of both Results and Efficacy sections. The correct table and paragraphs are given below.

**Table 1:** Demographic and baseline data (*n* = 9)

Variable	Mean (SD)	Range
Age	43.89 (7.36)	32–54
Age first homeless	35.22 (13.07)	15–50
Years homeless	7.33 (5.87)	1–22
Age first smoked	13.17 (2.98)	7.5–18
Years smoked	30.44 (9.37)	14–46
Number of cigarettes smoked	25.22 (8.24)	12–40
Carbon monoxide (ppm)	21.89 (14.41)	7–53
Fagerström	7.89 (1.27)	7–9
MPSS (Q1-7)*	18.11 (8.62)	7–31
MPSS (Q 8 and Q9)**	6.89 (2.85)	3–10
MPSS (Q10–12)***	4.56 (1.59)	3–8

## Results

Of 30 STA residents, 23 enrolled. In total, 14 recruited participants out of the 23 did not complete the intervention, leaving 9 participants who did. Two identified as women and seven identified as men. Study participants were aged between 32 and 54 years old (mean 43.89 years; SD 7.36 years), first became homeless between 15 and 50 years old (mean 35.22 years; SD 13.07 years) and had been homeless between 1 and 22 years (mean 7.33 years; SD 5.87 years). Study participants reported starting smoking between 7.5 and 18 years (mean 13.17 years; SD 2.98), having smoking histories between 14 and 46 years (mean 30.44; SD 9.37) and smoking between 12 and 40 cigarettes per day (mean 25.22; SD 8.24). At baseline, study participants measured between 7 and 53 ppm CO (mean 21.89; SD 14.41), between 7 and 9 in the *Fagerström* (mean 7.89; SD 1.27), between 7 and 31 in MPSS mood symptom composite score (Q1–7) (mean 18.11; SD 8.62), between 3 and 10 in MPSS “Urge to Smoke” and “Strength of Urges” composite score (Q8 and 9) (mean 6.89; SD 2.85) and between 3 and 8 in the MPSS physical symptom composite score (mean 4.56; SD 1.59) (see Table 1).

## Efficacy

Cigarette consumption was reported to decrease from a mean of 25.22 to 5.56 cigarettes (77.97% reduction). This self-reported decrease of cigarettes smoked was statistically significant (*p* < 0.001) (see Fig. 1). Mean carbon monoxide measurements decreased from 21.89 to 15.56 (28.93% reduction) with one participant measuring below the 5 ppm CO requirement to be considered a smoker. However, this decrease was not statistically significant (*p* = 0.079). The higher the number of cigarettes reported to be smoked at baseline, the lower the number of cigarettes decreased (*p* = 0.009; *r* =  − 0.807) (see Fig. 2). However, there was no statistically significant relationship between the number of cigarettes smoked at baseline and reductions in carbon monoxide (*p* = 0.531). Furthermore, there were no statistically significant relationships between years homeless, years smoked or “Quit” attempts and reductions in cigarette smoking.
